# Supplementary material for: Association between Wine Consumption with Cardiovascular Disease and Cardiovascular Mortality: A Systematic Review and Meta-Analysis
Source: Nutrients. 2023 Jun 17;15(12):2785. doi: 10.3390/nu15122785 (PMC10303697; doi:10.3390/nu15122785)
Supplement: Supplementary file 1 [file nutrients-15-02785-s001.zip › nutrients-2462588-supplementary.pdf]

Table S1. Complete search strategy for MEDLINE.

Adults<br>**OR**<br>"Young adults"<br>**OR**<br>"Elderly adults"<br>**OR**<br>"Older adults"<br>**OR**<br>"Adult population"<br>**OR**<br>"Adult subjects"	**AND**	Alcohol<br>**OR**<br>Wine<br>**OR**<br>"Alcohol consumption"<br>**OR**<br>"Wine consumption"	**AND**	CVD<br>**OR**<br>"Cardiovascular disease"<br>**OR**<br>"Coronary heart disease"<br>**OR**<br>"Heart failure"<br>**OR**<br>"Cardiovascular events"<br>**OR**<br>"Coronary artery disease"<br>**OR**<br>"Myocardial infarction"<br>**OR**<br>"Cardiovascular outcomes"<br>**OR**<br>Mortality<br>**OR**<br>"Cardiovascular mortality"<br>**OR**<br>"Cardiovascular death"	**AND**	Cohort<br>**OR**<br>"Case-control"<br>**OR**<br>"Longitudinal studies"<br>**OR**<br>"Prospective studies"

Table S2. Adjustments to the results of each study.

**Reference**	**Adjusted for**
Kaufman et al. 1985	Age, geographic region of the hospital, religion, marital status, years of education, cigarette smoking, history of drug treatment for hypertension, history of abnormal blood lipids, history of drug treatment for diabetes mellitus, body mass index, family history of myocardial infarction or stroke, personality score, and minutes of aerobic exercise per week.
Klatsky et al. 1986	Not adjusted.
Klatsky et al. 1990	Age, gender, race, body mass index, marital status and education.
Klatsky et al. 1992	Age.
Klatsky et al. 1993	Age, gender, race, smoking, body mass index, marital status, education, coffee and tea.
Gronbaek et al. 1995	Not adjusted.
Renaud et al. 1999	Age, smoking, education, body mass index and physical activity.
Theobald et al. 2000	Age, expected level of need for health services, total alcohol consumption, gender, body-mass index, to- bacco use and social class.
Gronbaek et al. 2000	Age, sex, smoking status, educational level, physical activity and body mass index.
Tavani et al. 2001	Age, education, parity, age at menopause, oral contraceptive use, family history of ovarian/breast cancer, body mass index, and energy intake (without alcohol calories).
Mukamal et al. 2003	Age, sex, race, apolipoprotein (APOE e4), status, educational attainment, income level, marital status, estrogen replacement therapy, current smoking, former smoking, diabetes, body mass index, total cholesterol level, atrial fibrillation, history of congestive heart failure, history of stroke, history of transient iscemic attack, kilocalories expended in daily activities, and consumption of other alcoholic beverages.
Marques-Vidal et al. 2004	Study center, age, cigarette smoking, hyperlipidemia, hypertension status, body mass index and diabetes.
Dorn et al. 2007	Age and education in years, race, BMI, smoking status (current, former, never) and menopausal status.
Burke et al. 2007	Cholesterol, blood pressure, waist girth, smoking habits and exercise.
Schröder et al. 2007	Age, total alcohol consumption, smoking, educational level, leisure-time physical activity, total cholesterol, LDL-cholesterol, HDL-cholesterol, diabetes, hypercholesterolemia drug treatment, and diagnosed hypertension.
Suadicani et al. 2008	Age.
Gémes et al. 2016	Age, sex, level of education, cohabiting, smoking, physical activity and body mass index.
Britton et al. 2016	Not adjusted.
Tverdal et al. 2017	Age and sex.
Ricci et al. 2018	Age at recruitment, body mass index, height, physical activity, smoking status and history of hypertension
Song et al. 2018	Age, sex, body mass index, race, smoking, education and exercise.
Panagiotakos et al. 2019	Age, sex, smoking (years), physical activity (active / inactive), BMI, education in years of school, MedDietScore, alcohol quantity, history of hypertension, diabetes mellitus and hypercholesterolemia.
Schutte et al. 2020	Sex, age and body mass index.
Maugeri et al. 2020	Number of drinking days, age, gender, educational level, and employment status.
Schutte et al. 2021	Age, body mass index (BMI), sex, smoking, systolic blood pressure, diagnosis of diabetes and Townsend deprivation index.

Table S3. Risk of bias assessment table using Quality Assessment Tool for Observational Cohort and Cross-Sectional Studies.

References	Items														Quality
	**1**	**2**	**3**	**4**	**5**	**6**	**7**	**8**	**9**	**10**	**11**	**12**	**13**	**14**	
Klatsky et al. 1986	Y	Y	Y	Y	N	Y	Y	Y	Y	N	Y	NR	Y	Y	Good
Klatsky et al. 1990	Y	Y	Y	Y	N	Y	Y	Y	Y	N	Y	NR	Y	Y	Good
Klatsky et al. 1992	Y	Y	Y	Y	N	Y	Y	Y	Y	N	Y	NR	Y	Y	Good
Klatsky et al. 1993	Y	Y	Y	Y	N	Y	Y	Y	Y	N	Y	NR	Y	Y	Good
Gronbaek et al. 1995	Y	Y	Y	Y	Y	Y	Y	Y	Y	Y	Y	Y	Y	Y	Good
Renaud et al. 1999	Y	Y	Y	Y	N	Y	Y	Y	Y	N	Y	NR	Y	Y	Good
Theobald et al. 2000	Y	Y	Y	Y	N	Y	Y	Y	Y	N	Y	NR	Y	Y	Good
Gronbaek et al. 2000	Y	Y	Y	Y	N	Y	Y	Y	Y	N	Y	NR	Y	Y	Good
Mukamal et al. 2003	Y	Y	Y	Y	N	Y	Y	Y	Y	N	Y	NR	Y	Y	Good
Marques-Vidal et al. 2004	Y	Y	Y	Y	N	Y	Y	Y	Y	Y	Y	NR	Y	Y	Good
Burke et al. 2007	Y	Y	Y	Y	Y	Y	Y	Y	Y	N	Y	NR	Y	Y	Good
Suadicani et al. 2008	Y	Y	Y	Y	N	Y	Y	Y	Y	N	Y	NR	Y	Y	Good
Gémes et al. 2016	Y	Y	Y	Y	N	Y	Y	Y	Y	N	Y	NR	Y	Y	Good
Britton et al. 2016	Y	Y	Y	Y	Y	Y	Y	Y	Y	N	Y	NR	Y	Y	Good
Tverdal et al. 2017	Y	Y	Y	Y	N	Y	Y	Y	Y	N	Y	NR	Y	Y	Good
Ricci et al. 2018	Y	Y	Y	Y	N	Y	Y	Y	Y	N	Y	NR	Y	Y	Good
Song et al. 2018	Y	Y	Y	Y	Y	Y	Y	Y	Y	N	Y	NR	Y	Y	Good
Panagiotakos et al. 2019	Y	Y	Y	Y	Y	Y	Y	Y	Y	N	Y	NR	Y	Y	Good
Schutte et al. 2020	Y	Y	Y	Y	Y	Y	Y	Y	Y	N	Y	NR	Y	Y	Good
Maugeri et al. 2020	Y	Y	Y	Y	Y	Y	Y	Y	Y	N	Y	NR	Y	Y	Good
Schutte et al. 2021	Y	Y	Y	Y	Y	Y	Y	Y	Y	N	Y	NR	Y	Y	Good

1. Was the research question or objective in this paper clearly stated?; 2. Was the study population clearly specified and defined?; 3. Was the participation rate of eligible persons at least 50%?; 4. Were all the subjects selected or recruited from the same or similar populations (including the same time period)? Were inclusion and exclusion criteria for being in the study prespecified and applied uniformly to all participants?; 5. Was a sample size justification, power description, or variance and effect estimates provided?; 6. For the analyses in this paper, were the exposure(s) of interest measured prior to the outcome(s) being measured?; 7. Was the timeframe sufficient so that one could reasonably expect to see an association between exposure and outcome if it existed?; 8. For exposures that can vary in amount or level, did the study examine different levels of the exposure as related to the outcome (e.g., categories of exposure, or exposure measured as continuous variable)?; 9. Were the exposure measures (independent variables) clearly defined, valid, reliable, and implemented consistently across all study participants?; 10. Was the exposure(s) assessed more than once over time?; 11. Were the outcome measures (dependent variables) clearly defined, valid, reliable, and implemented consistently across all study participants?; 12. Were the outcome assessors blinded to the exposure status of participants?; 13. Was loss to follow-up after baseline 20% or less?; 14. Were key potential confounding variables measured and adjusted statistically for their impact on the relationship between exposure(s) and outcome(s)?; N: no; NR: not reported; Y: yes.

Table S4. Risk of bias assessment table using Quality Assessment Tool for Case-Control Studies

References	Items												Quality
	**1**	**2**	**3**	**4**	**5**	**6**	**7**	**8**	**9**	**10**	**11**	**12**	
Kaufman et al. 1985	Y	Y	Y	Y	Y	Y	N	Y	Y	Y	N	Y	Good
Tavani et al. 2001	Y	Y	Y	Y	Y	Y	Y	Y	Y	Y	NR	Y	Good
Dorn et al. 2007	Y	Y	Y	Y	Y	Y	Y	Y	Y	Y	Y	Y	Good
Schröder et al. 2007	Y	Y	Y	Y	Y	Y	Y	Y	Y	Y	NR	Y	Good

1.Was the research question or objective in this paper clearly stated and appropriate?; 2. Was the study population clearly specified and defined?; 3. Did the authors include a sample size justification?; 4. Were controls selected or recruited from the same or similar population that gave rise to the cases (including the same timeframe)?; 5. Were the definitions, inclusion and exclusion criteria, algorithms or processes used to identify or select cases and controls valid, reliable, and implemented consistently across all study participants?; 6. Were the cases clearly defined and differentiated from controls?; 7. If less than 100 percent of eligible cases and/or controls were selected for the study, were the cases and/or controls randomly selected from those eligible?; 8. Was there use of concurrent controls?; 9. C If matching was used, did the investigators account for matching during study analysis?; 10. Were the measures of exposure/risk clearly defined, valid, reliable, and implemented consistently (including the same time period) across all study participants?; 11. Were the assessors of exposure/risk blinded to the case or control status of participants?; 12. Were key potential confounding variables measured and adjusted statistically in the analyses?; N: no; NR: not reported; Y: yes.

Table S5. Sensitivity analysis.

CHD

**Reference**	**RR**	**LL**	**UL**
Kaufman et al. 1985	0,7824	0,635	0,814
Klatsky et al. 1986	0,722	0,631	0,813
Gronbaek et al. 2000	0,741	0,649	0,833
Tavani et al. 2001	0,744	0,652	0,835
Mukamal et al. 2003	0,724	0,632	0,815
Mukamal et al. 2003	0,718	0,624	0,812
Marques-Vidal et al. 2004	0,713	0,618	0,808
Marques-Vidal et al. 2004	0,742	0,653	0,831
Dorn et al. 2007	0,734	0,642	0,826
Gémes et al. 2016	0,724	0,626	0,820
Song et al. 2018	0,719	0,622	0,815
Ricci et al. 2018	0,716	0,616	0,816
Schutte et al. 2021	0,726	0,626	0,826

CVD

**Reference**	**RR**	**LL**	**UL**
Renaud et al. 1999	0,750	0,551	0,950
Burke et al. 2007	0,830	0,691	0,969
Tverdal et al. 2017	0,767	0,581	0,953
Panagiotakos et al. 2019	0,808	0,650	0,966
Maugeri et al. 2020	0,748	0,549	0,947
Schutte et al. 2021	0,730	0,596	0,865

Cardiovascular mortality

**Reference**	**RR**	**LL**	**UL**
Klatsky et al. 1992	0,713	0,560	0,866
Klatsky et al. 1992	0,678	0,525	0,831
Klatsky et al. 1993	0,702	0,537	0,866
Gronbaek et al. 1995	0,737	0,593	0,881
Theobald et al. 2000	0,724	0,578	0,869
Suadicani et al. 2008	0,679	0,524	0,835
Britton et al. 2016	0,664	0,558	0,770

Table S6. Meta-regression according to mean age, percentage of female and follow-up of wine exposure on ischaemic heart disease, cardiovascular disease and cardiovascular mortality.

	Coefficient	95%ICs	P value
**Ischaemic heart disease**			
Mean age	-0.011	-0.046, 0.024	0.508
Percentage of female	-0.007	-0.007, 0.006	0.813
Follow-up	-0.007	-0.016, 0.015	0.917
Current smokers	-0,007	-0.016, 0.001	0.081
**Cardiovascular disease**			
Mean age	-0.005	-0.075, 0.065	0.833
Percentage of female	0.001	-0.010, 0.012	0.779
Follow-up	-0.015	-0.153, 0.122	0.743
Current smokers	-0,008	-0.017, 0,000	0,049
**Cardiovascular mortality**			
Mean age	0.040	-0.020, 0.099	0.148
Percentage of female	0.001	-0.014, 0.017	0.833
Follow-up	0.023	-0.026, 0.073	0.276
Current smokers	-0.002	-0.031, 0.027	0.870
